# Design and evaluation of leadership development program using co-creation approaches for an athlete leadership group among elite high school rugby teams

**DOI:** 10.3389/fspor.2026.1770549

**Published:** 2026-03-06

**Authors:** Toshiaki Hirose, Yasutaka Ojio, David Lavallee, Naohiko Kohtake

**Affiliations:** 1 Graduate School of System Design and Management, Keio University, Kanagawa, Japan; 2 Graduate School of Arts and Sciences, The University of Tokyo, Tokyo, Japan; 3 Department of Health, Sport and Wellbeing, Abertay University, Dundee, United Kingdom

**Keywords:** athlete leadership group, co-creation approaches, collective reflection, cyclical intervention design, shared leadership, talent development, transformational leadership, youth rugby

## Abstract

**Introduction:**

Captains and athlete leaders in Japanese high school rugby teams are often expected to lead without formal leadership training, resulting in role ambiguity and uneven psychological burden. This study designed and evaluated the feasibility and acceptability of a fully online, theory-driven leadership development program using co-creation approaches for an athlete leadership group.

**Methods:**

Thirty-three leaders (8 captains, 25 athlete leaders) from eight competitive high school rugby teams completed the program. Two delivery patterns were identified: a brief intervention group (*n* = 13), which completed a five-step program, and an intervention group (*n* = 20), which engaged in repeated cycles including action implementation, monitoring, collective reflection, and replanning. A mixed-methods evaluation combined stepwise process ratings with semi-structured interviews of leaders and coaches.

**Results:**

Participants perceived improved role clarity, ownership of leadership behaviors, enhanced communication, and more effective distribution of responsibilities. Moreover, participants in the intervention group which conducted repeated cycles reported to exhibit more stable behavioral enactment, greater peer involvement, and more relational leadership practices. Captains reported reduced psychological burden as leadership became more shared, and coaches observed enhanced autonomy and player-driven coordination.

**Discussion:**

These findings suggest that this program was feasible and acceptable. Brief delivery initiated meaningful insights and short-term behavioral change, while the sustained cycle produced broader impact within teams. Earlier-season, longer-duration implementation may further support youth rugby talent development and the cultivation of sustainable shared leadership structures.

## 1 Introduction

Leadership has become a central construct in contemporary sport science, with growing evidence linking effective leadership to both enhanced team performance and athlete well-being (1, 2). In sport, leadership is typically conceptualized as a dynamic process of social influence within a group that is directed toward collective goals (3). Leadership provided by coaches, who exert primary influence on sports teams as formal leaders (4), has been a natural focus of research (5, 6). In addition to coaches, recent research has highlighted the complementary contributions of both formal (e.g., captains) and informal athlete leaders, who influence players through both task and social channels (7, 8). Among available frameworks, transformational leadership (TFL) has attracted particular attention because of its potential to enhance coordinated team functioning (6, 9, 10). For example, TFL has consistently been associated with higher athlete motivation, performance, and well-being (11–13). Captains' TFL behaviors have been linked to task cohesion, whereas athlete leaders' TFL may preferentially foster social cohesion (14, 15). These findings align with the concept of shared leadership, in which leadership responsibilities are distributed across multiple individuals (16, 17). In some cases, athlete leaders are regarded as an informal group of peer leaders; herein, we define them as formally designated vice-captains and unit or positional leaders (excluding the captain). Team captains and athlete leaders are players formally designated by the coaching staff or team to support both tactical and psychological aspects of team functioning. The captain typically acts as the symbolic leader, representing the team externally and serving as a liaison between coaches and players (18, 19). Athlete leaders complement the captain by providing role-based support, such as facilitating peer communication, offering tactical guidance, and promoting team cohesion during practices and matches.

Rugby is a sport in which coaches' intervention during matches is minimal, placing particular emphasis on in-game decision-making by captains and athlete leaders. Furthermore, the diverse range of positions on the field necessitates multiple leaders. Therefore, a shared understanding of team strategy, interpersonal relationships, and mutual support among players becomes crucial. In line with achievement goal theory (20), we focused on athletes' shared perceptions of the team's motivational climate (i.e., the extent to which effort, learning, and cooperation vs. social comparison are emphasized). Motivational climate is a key contextual factor shaping team dynamics, defined as athletes' perceptions of the team learning environment. It is typically distinguished into task-involving climate (focused on effort and personal growth) or ego-involving climate (focused on outperforming others) (21). TFL has been shown to foster task-involving climate, which in turn supports team cohesion and sustained effort (22–24). Teams with robust leadership groups tend to report more task-involving and less ego-involving climates (25). Shared leadership and TFL have been linked to stronger task-involving climate and ultimately to greater team resilience in elite rugby national teams (26, 27). Our previous findings from elite youth Japanese rugby teams similarly suggest that the TFL of head coaches, captains, and athlete leaders exerts distinct yet complementary effects on motivational climate and team resilience (28). Consequently, it is necessary to include not only the captain but also athlete leaders as an athlete leadership group to enhance task-involving climate and reduce ego-involving climate. In fact, Australian Football League teams and a rugby team in New Zealand have utilized athlete leadership groups that integrate captains and vice-captains (29). This approach indicates that coaches delegate leadership responsibilities to leaders (30) and leadership groups represent more horizontal mechanisms in which decision-making is distributed among members. Cotterill et al. (31) also showed that the use of leadership groups to support the captain and spread the leadership load in elite UK rugby team is increasing, and similar trends can be observed even among Japan's youth elite-level teams.

Youth sport represents a highly formative period in which athletes undergo rapid physical, psychological, and social development, making this stage particularly influential for shaping long-term sport participation and talent outcomes (32, 33). Psychological skills such as motivation, resilience, and leadership are especially malleable during adolescence, and appropriate developmental environments have been shown to play a decisive role in athletes' future performance trajectories (34). Furthermore, the youth stage represents a key transition and selection point in many talent development pathways. High-quality motivational climates and shared leadership structures during adolescence have been linked to enhanced team functioning, performance, and psychological well-being (35, 36). The leadership and psychosocial skills developed during adolescence are widely recognized as transferable to school, social relationships, and later vocational contexts (34, 37, 38). Understanding how these competencies emerge and can be supported in youth sport holds substantial applied and theoretical significance. At the same time, youth athletes are exposed to considerable performance pressures, role ambiguity, and interpersonal challenges, which can increase the risk of burnout, dropout, or maladaptive psychosocial outcomes if not properly supported (39, 40). However, research consistently shows that youth leaders receive little formal leadership training despite being assigned substantial responsibilities (16, 41, 42). Coaches often do not possess sufficient knowledge of how to develop leadership skills and abilities of their leaders (43). Captains and athlete leaders are frequently selected without transparent criteria and must learn their roles through observation and trial-and-error, often with limited guidance (42, 44, 45). As a result, many leaders report uncertainty about role expectations and heightened psychological burden, in particular, this psychological burden tends to be greater for captains (35, 46–49).

In previous studies, most interventions were initially coach-centered [e.g., TFL workshops; (5, 50)] and later shifted toward a focus on captains as individual leader (43, 46). This progression highlights the importance of ongoing support and of enhancing a sense of responsibility and ownership. More recent programs have begun to incorporate shared-leadership principles by engaging broader leadership groups and the team as a unit. For example, Voight (51) combined regular online and on-site activities for captains, assistant captains and apprentices using assessment tools and iterative feedback to improve leadership and team communication across a season. Participants reported perceived benefits to team cohesion, peers' performance, and their own leadership skills. Fransen et al. (52) used the 5R approach (Readying, Reflecting, Representing, Realizing, Reporting) to build collective identity and social network analysis to distribute and enhance leadership. Building on these works, scholars have emphasized the importance of aligning leadership styles with athletes' developmental needs and of providing regular, practical support opportunities. However, many programs have been constrained by face-to-face delivery, which limits scalability.

**Figure 1:**
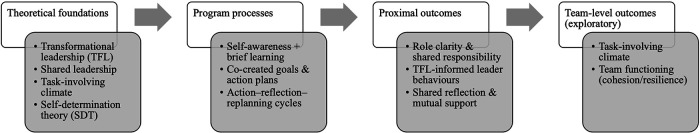
Brief conceptual model of the program’s theoretical integration and logic. The diagram summarizes how TFL, shared leadership, task-involving climate, and SDT inform the program processes (self-awareness/brief learning, co-created action planning, and action–reflection–replanning cycles) and the intended proximal and exploratory team-level outcomes.

Online leadership programs for adolescent athletes have gained increasing attention. For example, the NFHS Captain's Course has been shown to enhance leadership knowledge and promote self-reflection among high school athletes (53). Related work suggests that combining online leadership education with offline coaching can support the translation of learning into practice (54), and online workshops have been identified as a feasible and non-invasive approach to youth leadership development (55). However, existing evaluations rely largely on self-report outcomes, focus primarily on formally designated leaders as individuals (e.g., captains), and drawing on research-based leadership concepts, few explicitly integrate established theories to structure leadership learning within athlete leadership groups. Taken together, the current body of research offers promising foundations for athlete leadership development but also reveals critical gaps: few examine theory-driven leadership processes, and few programs explicitly target an athlete leadership group (captain and athlete leaders), more specifically, this group is rarely treated as the primary unit of change, and few interventions embed structured cycles of role clarification, co-created goal setting and action planning, and collective reflection within real teams. An online, co-created format is particularly relevant in adolescent school sport because leadership roles are highly context-specific and often ill-defined, and repeated face-to-face delivery is frequently constrained by timetabling and limited staff resources. These gaps highlight needs for research investigating online leadership development within real sport teams, particularly in adolescent populations where access to an athlete leadership group education is limited. In the present study, we intentionally scheduled the program late in the competitive season, when cohesive functioning is paramount, to respond to evidence that longer team tenure may foster ego-involving climates (56) by explicitly targeting attitudinal and behavioral change among athlete leaders. To summarize the program's theoretical integration and logic, Figure 1 presents a brief conceptual model linking the theories, program processes, and intended outcomes.

The primary aim was to provide a TIDieR-guided, replicable description of the design and implementation of an online co-created leadership development program for adolescent athletes in sport (57). The secondary aim was to examine feasibility and acceptability as the main outcomes and to explore process-level signals of change through a mixed-methods evaluation combining quantitative indicators and qualitative insights. Clarifying these aspects holds significance for advancing the development of youth athlete leaders both theoretically and practically.

## 2 Methods

### 2.1 Study design

This study used a co-creation–based mixed-methods feasibility design. In line with participatory and shared leadership principles, captains and athlete leaders collaborated with the facilitator (first author) to clarify roles, identify leadership challenges, and co-creation goal setting and action plans for their own teams. The program facilitator was also the first author of the study. This dual role was necessary due to the specialized expertise required to design and deliver a theory-driven, co-creation–based leadership intervention within elite youth sport settings. To mitigate potential bias, standardized materials and structured procedures were used across teams, and qualitative data analysis involved another researcher (second author). In addition, coaches were interviewed to provide an external perspective and to triangulate participants' accounts of feasibility and perceived program effects. Specifically, facilitation followed standardized content and a structured agenda, emphasizing reflection and co-creation rather than evaluation. Interviews used a semi-structured guide with consistent core prompts, were audio-recorded and transcribed verbatim, and coach interviews supported triangulation. The second author contributed to coding and theme development, with discrepancies resolved through discussion; however, some influence of the dual role by the researcher and the facilitator cannot be fully eliminated in applied feasibility work.

#### 2.1.1 Co-creation approaches

Because roles within an athlete leadership group were unclear and information sharing was insufficient, we adopted co-creation approaches in which leaders jointly designed their own leadership behaviors and action plans. Co-creative leadership emphasizes a collective process of integrating multiple perspectives to build a shared, systemic view of the team (58). In this program, leaders first engaged in self-awareness (43, 59, 60) and theoretical learning, and then used dialogue with the facilitator and other leaders to co-create concrete leadership goal and actions. The action plans were visualized and shared within the athlete leadership group and with the facilitator.

#### 2.1.2 Theory-driven approaches

This study employed a theory-driven online program informed by TFL, motivational climate frameworks, self-determination theory (SDT) (61), and shared leadership models, which align with (5, 50, 52, 62, 63) and emphasize a common set of developmental principles: collaborative goal setting, role clarification, supportive relatedness, and ongoing structured reflection (64). The intervention integrates six core components of TFL with three task-involving climate dimensions (improvement, effort, relatedness support) and, within an SDT framework, emphasizes autonomy, competence, and relatedness by having leaders co-create role-specific action plans, provide reciprocal support, and reflect together on implementation. Contemporary recommendations further suggest that coaches and sport psychologists provide individualized, developmentally appropriate tasks that align with Generation-*Z* values, including closeness, commitment, and co-orientation within the team environment (65, 66).

#### 2.1.3 Cycle of action implementation, collective reflection, and replanning goal and action

Each co-created leadership action was implemented in practice with the aim of enhancing the task-involving climate and team performance. The actions taken were shared via a messaging app and a cloud-based survey system. The messaging app was used on a daily basis and included groups for the facilitator and the athlete leadership group, where collective reflection took place. Regarding the cloud-based survey system, responses were shared with the facilitator as background information for the weekly online video conferences. Using this information and through dialogue with leaders during the online video conferences, the athlete leadership group determined subsequent goal setting and action plans. These action–reflection–replanning cycles were implemented continuously throughout the intervention.

### 2.2 Participants and procedures

**Figure 2:**
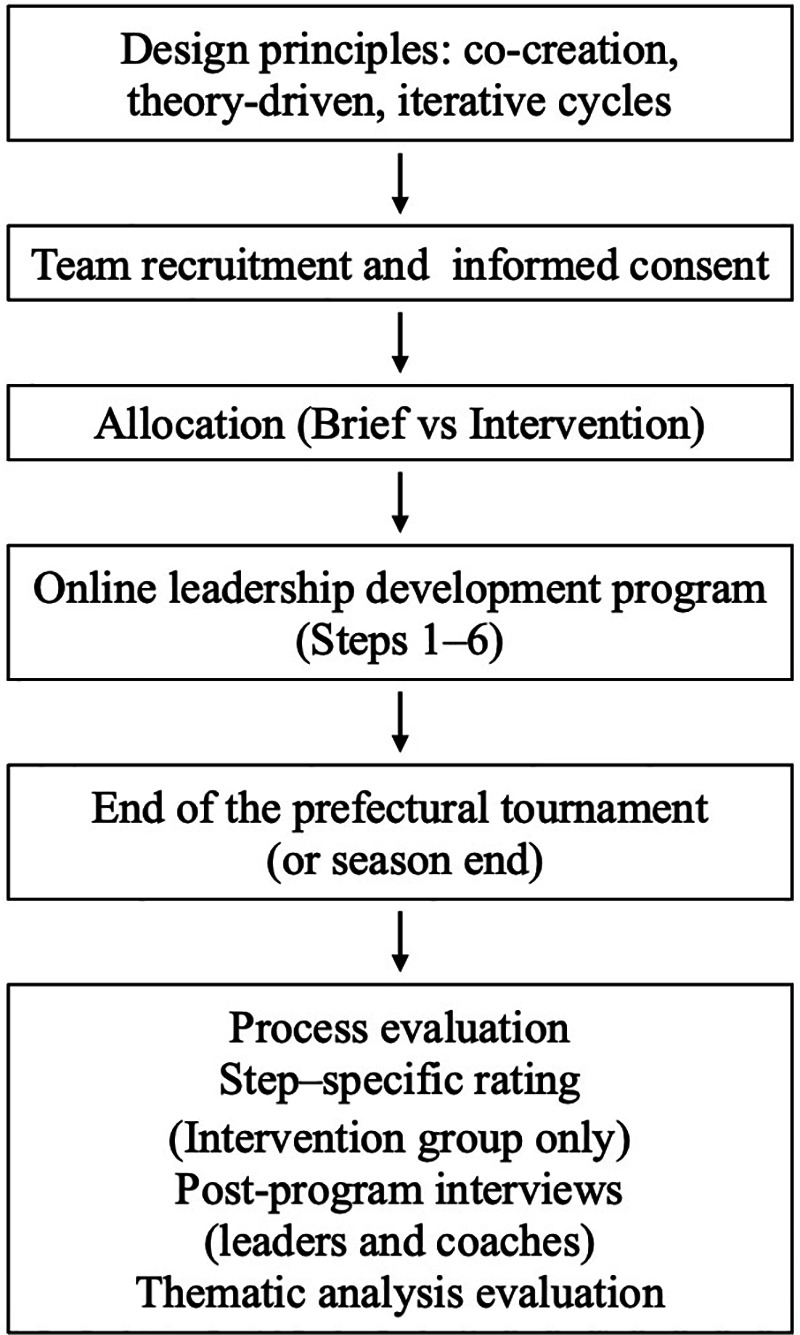
Research workflow. This figure illustrates the research flow. Teams were recruited and informed consent was obtained, after which participants followed one of two exposure patterns (a brief intervention group or an intervention group). The program consisted of six steps. The brief intervention group completed the program up to Step 5, whereas the intervention group repeatedly engaged in Steps 5 and 6 until the end of the season. Evaluation at each step for intervention group and post-program interviews with leaders and coaches were conducted, and the program was evaluated through process indicators and thematic analysis.

33 male high school rugby players (captains = 8; athlete leaders = 25) from eight nationally competitive programs took part during the period from 12 October to 18 November 2024. Schools were recruited through existing professional networks with national-level coaches and sport science researchers. This recruitment approach may have introduced selection bias (e.g., teams more open to leadership development and research participation). The participating schools were in five regions of Japan (Kanto, Kansai, Hokuriku, Shikoku, and Kyushu), providing geographical dispersion, and included three public and five private high schools. All teams had finished within the top eight of their respective prefectural championships in 2024. Coaches received a comprehensive verbal and written briefing and provided institutional consent for team participation. Individual written informed consent was obtained from each athlete and his legal guardian. The study protocol was approved by the Ethics Committee of the Graduate School of System Design and Management, Keio University (Approval No. SDM-2024-E036). The overall study workflow is summarized in Figure 2.

### 2.3 Intervention contents

**Figure 3:**
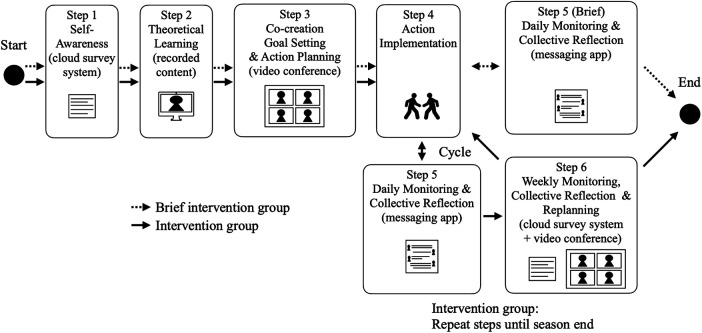
Study design and intervention process of the leadership development program. This figure presents the study design and procedural flow of the online leadership development program, illustrating differences between the brief intervention group and the intervention group. Following recruitment and allocation, participants completed a structured six-step program beginning with self-awareness and theoretical learning, followed by co-creation-based goal setting and action planning. After action implementation, both groups engaged in daily monitoring and collective reflection using a messaging application. The brief intervention group completed the program at this stage, whereas the intervention group continued iterative cycles of daily monitoring and weekly collective reflection and replanning using cloud-based surveys and video conferencing until the end of the season. These cyclical processes were designed to support sustained leadership practice, reflection, and adaptive goal adjustment over time.

Reporting of the intervention followed the TIDieR checklist (Table 1). Figure 2 provides a flow chart of the Methods (recruitment/consent, exposure patterns, intervention steps, and evaluation procedures). Figure 3 provides an at-a-glance overview of the six-step program flow and the iterative cycle (Steps 4–6). The program targeted leadership behaviors among the athlete leadership group and was underpinned by TFL, task-involving climate, SDT, and shared leadership. The program was delivered through six iterative components: (1) self-awareness, (2) theoretical learning, (3) co-creation goal setting and action planning, (4) action implementation, (5) daily monitoring and collective reflection, and (6) weekly monitoring, collective reflection and replanning goal and action, using a blend of synchronous (online video conference system) sessions and asynchronous (recorded video, messaging app, cloud-based survey system) activities.

**Table 1:** TIDieR checklist for the leadership development program.

TIDieR Item	Contents
1. Intervention name	Online Leadership Development Program for an Athlete Leadership Group
2. Why (rationale and theory)	Underpinned by transformational leadership (TFL), task-involving climate, self-determination theory (SDT), and shared leadership framework. The program aimed to strengthen the capacity of captains and athlete leaders to enhance TFL and cultivate task-involving climate.
3. What—materials	Recorded video (covering six TFL components and three task-involving climate facets), self-awareness questionnaire, online video conference system slide deck, cloud-based survey system for weekly monitoring and messaging app for monitoring, coordination and leader support, and semi-structured interview guide.
4. What—procedures	Six-step sequence: (1) Self-awareness, (2) Theoretical learning, (3) Co-creation goal setting and action planning, (4) Action implementation, (5) Daily monitoring and collective reflection, and (6) Weekly monitoring, collective reflection and replanning goal and action, followed by a summative quantitative and interview-based evaluation.
5. Who provided	Program facilitator and implementer: Toshiaki Hirose. Program design/oversight: research team (sport psychology/mental health). Interview facilitation: lead interviewer = licensed clinician–researcher (more than 10 years of experience in athlete mental health/leadership); co-interviewer = trained graduate-student interviewer (near-peer age).
6. Where	Entirely online (online video conferences for synchronous sessions; the messaging app and the cloud-based survey system for asynchronous communication).
7. When and how much	Program window: from initial session to the end of each team's 2024 prefectural (or national, for one team) tournament. Brief intervention group: 1 Response to self-awareness questionnaire via the cloud-based survey system; 1 online video conference (35mins); an average of 2 messaging app exchanges; no cloud-based survey system entries. Intervention group: 1 Response to self-awareness questionnaire via the cloud-based survey system; an average of 2.8 online video conference (35mins); an average of 7.2 messaging app exchanges; an average of 2.4 weekly monitoring submissions per leader via the cloud-based survey system.
8. How (mode of delivery)	Hybrid online delivery: synchronous online video conferences plus asynchronous recorded video, daily monitoring and collective reflection via the messaging app, and self-awareness questionnaire and weekly monitoring via the cloud-based survey system.
9. Tailoring	Scheduling adapted to each team's training and competition calendar; extended activities for the team advancing to the national tournament.
10. Modifications	No substantive protocol changes; minor timing adjustments to avoid competition conflicts.
11. Fidelity—planned	Anticipated variability in engagement with Steps 4–6 due to tournament progression.
12. Fidelity—actual	Attendance logs (online video conference system), messaging counts (messaging app), and self-monitoring submissions (cloud-based survey system) documented exposure; group-level dosage is summarized in Table 2.

### 2.4 Intervention groups

Two exposure patterns emerged due to differences in competition schedules; these were treated as contextual factors when interpreting behavior change and quality of leadership development, particularly the implementation of Steps 4–6 (Figure 3). Figure 3 also visually contrasts the brief (Step 5 only) and intervention (repeated Steps 4–6) exposure patterns.

**Figure 4:**
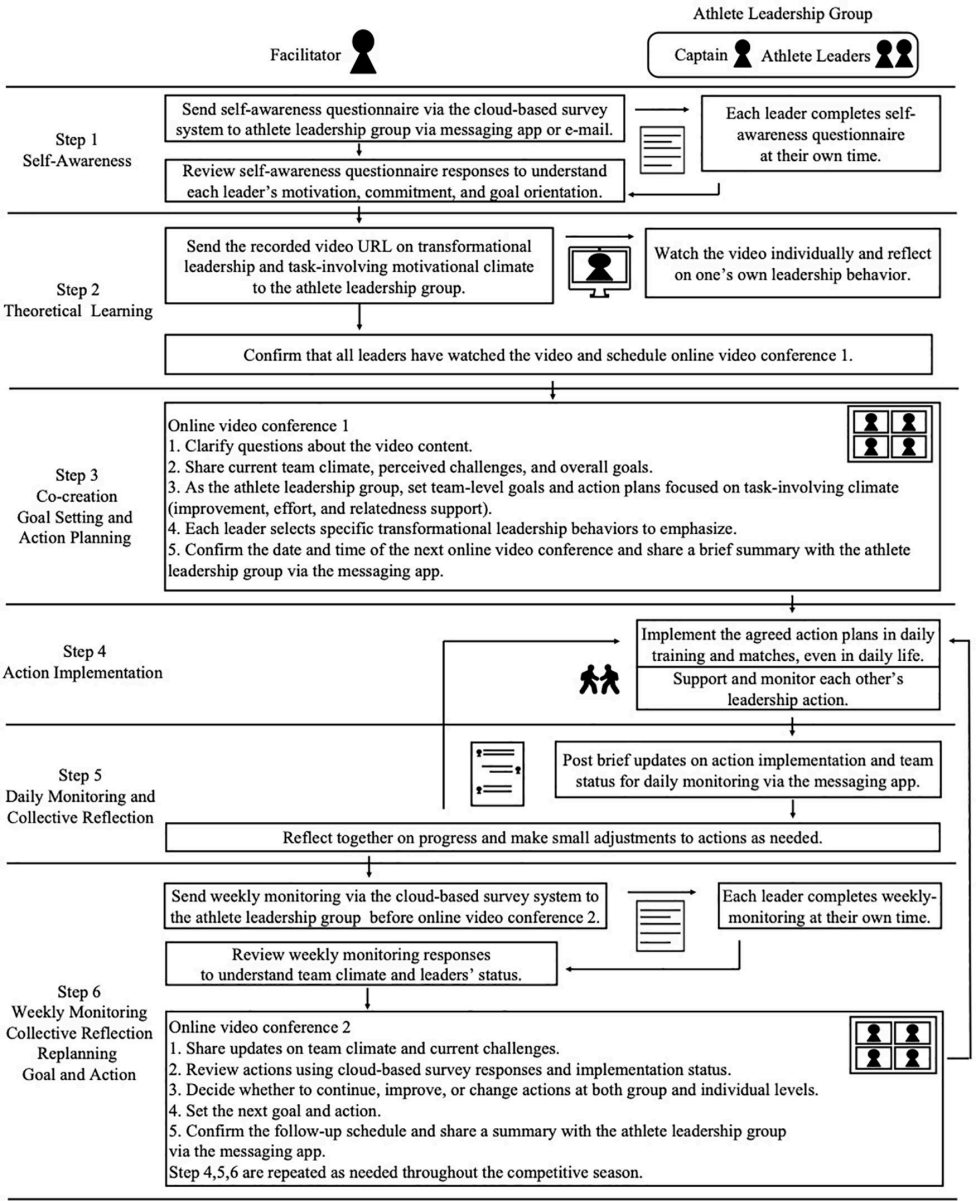
Detail intervention process of the leadership development program. Diagram showing a six-step leadership development program involving a facilitator and an athlete leadership group, including captain and athlete leaders. The process includes self-awareness questionnaires, video-based theoretical learning, online group discussions for goal setting and action planning, implementation of leadership actions, and daily and weekly monitoring with collective reflection, replanning the goal and action with steps 4–6 repeated across the competitive season.

Brief intervention group: Team completed only the cycle of Steps 4,5 before proceeding to the post-program interview (i.e., one planning and one implementation check before tournament exit, with no Step 6). Typical engagement comprised one response to self-awareness questionnaire via the cloud-based survey system, one 35-min online video conference for co-creation goal setting and action planning (Figure 4: Online video conference 1), approximately two instances of daily monitoring via the messaging app exchanges, and no weekly monitoring via the cloud-based survey system.

Intervention group: Team completed multiple cycles of Steps 4–6 across the observation window. Typical engagement comprised one response to self-awareness questionnaire via the cloud-based survey system, one 35-min online video conference (Figure 4: Online video conference 1), about 7.2 instances of daily monitoring via the messaging app exchanges, approximately 2.4 weekly monitoring submissions per person via the cloud-based survey system, and approximately 1.8 additional ∼35-min online video conference (Figure 4: Online video conference 2) (Table 2).

**Table 2:** Exposure to program components in the brief intervention group and the intervention group.

Step	Brief intervention group	Intervention group
1	Same as intervention group (Self-awareness questionnaire via cloud-based survey system)	Same as brief intervention group
2	Same as intervention group (Theoretical learning via recorded video)	Same as brief intervention group
3	Same as intervention group One 35-min online video conference for goal setting and action planning (see Figure 4: online video conference 1)	Same as brief intervention group
4	Action plans implemented within each team across multiple cycles of steps 4,5	Action plans implemented within each team across multiple cycles of steps 4,5 and steps 4–6
5	Frequency of daily monitoring via the messaging app: approximately 2 posts per the athlete leadership group	Frequency of daily monitoring via the messaging app: approximately 7.2 posts per the athlete leadership group
6	No weekly monitoring via cloud-based survey system; no additional online video conferences	Approximately 2.4 weekly monitoring submissions per leader via the cloud-based survey system and approximately 1.8 additional 35-min online video conferences (Figure 4: online video conference 2)

### 2.5 Evaluation of the study

#### 2.5.1 Evaluation of step-specific process

**Figure 5:**
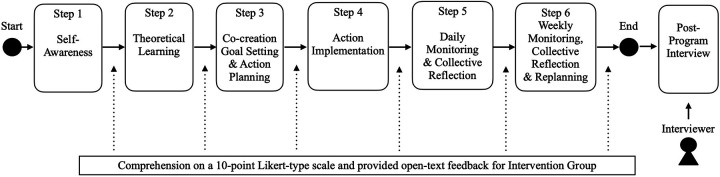
Evaluation approaches. Flowchart illustrating a six-step intervention process progressing from self-awareness and theoretical learning to co-creation goal setting, action implementation, daily monitoring and reflection, and weekly monitoring with reflection and replanning, followed by a post-program interview. Beneath the flowchart, a note indicates that participant comprehension was assessed using a 10-point Likert-type scale with open-ended feedback for a subset of the intervention group (*n* = 7).

The timing of process evaluation and post-program interviews is also depicted in Figure 2 (see Figure 5 for additional detail). At the conclusion of each program step, seven participants (captains = 2; athlete leaders = 5; out of 20 intervention group members) rated their comprehension on a 10-point Likert-type scale and provided open-text feedback (Figure 5). We computed descriptive statistics for perceived utility and used qualitative comments to identify actionable refinements to content and delivery.

#### 2.5.2 Post-program interviews

Following program completion, semi-structured one-on-one online interviews (Figure 5) were conducted with three captains, four athlete leaders and two coaches in the brief intervention group, and five captains, ten athlete leaders and five coaches in the intervention group. Interviews with coaches were also conducted to triangulate feasibility information. Interviews were facilitated by the lead interviewer or co-interviewer, lasted approximately 30 min each, and were conducted between 12 October and 1 December 2024. The guide covered five domains: (i) perceived effects, (ii) memorable experiences, (iii) personal learning, (iv) challenges encountered, and (v) future prospect. Example prompts included: (i) “Did you notice any positive effects of this program?”, (ii) “What kind of impact did you feel from this program?”, (iii) “What did you learn from this program?”, (iv) “What challenges did you encounter with this program?”, and (v) “How would you like this program to be utilized by juniors and others?”. All interviews were conducted online, audio-recorded, and transcribed verbatim.

#### 2.5.3 Thematic analysis

An inductive thematic analysis (67) was conducted in five phases: (i) meaning-unit extraction, (ii) open coding, (iii) category formation, (iv) theme integration, and (v) selection of illustrative quotations. Initial coding was conducted by the first author, and the second author reviewed the codebook and a subset of coded excerpts; disagreements were resolved through discussion to refine themes. An audit trail of analytic decisions was maintained. Coach interviews were used primarily to corroborate and elaborate player-reported themes and to explore how the program could support collaboration between leaders and coaches (68).

## 3 Results

### 3.1 Descriptive statistics

All 33 enrolled leaders completed the study. The brief intervention group comprised three teams, with three captains and ten athlete leaders. Captains' mean age in this group was 16.89 (SD = 0.10), with 7.97 years (SD = 0.10) of rugby experience and 2.50 years (SD = 0.08) of tenure on their current team. Athlete leaders in this group averaged 16.94 years of age (SD = 0.54), with 6.83 years (SD = 5.34) of rugby experience and 2.19 years (SD = 0.46) of tenure. The intervention group comprised five teams, with five captains and fifteen athlete leaders. Captains' mean age was 17.27 years (SD = 0.21), with 9.83 years (SD = 4.35) of rugby experience and 2.52 years (SD = 0.07) of tenure on their current team. Athlete leaders averaged 16.98 years of age (SD = 0.28), 9.97 years (SD = 4.66) of rugby experience, and 2.53 years (SD = 0.11) of tenure.

### 3.2 Step-specific process outcomes

Process-evaluation ratings were available for seven leaders who completed all stepwise assessments. On a 10-point Likert-type scale, mean scores ranged from 7.86 to 9.57, indicating consistently high acceptability. Overall, step-level ratings are summarized in Table 3.

**Table 3:** Program step–specific process evaluation ratings.

Step	Item	Mean score
Step 1 (Self-awareness)	Understanding of the questionnaire process	9
	Clarity of question content	8.86
	Ease of using self-awareness questionnaire via the cloud-based survey system	9.86
Step 2 (Theoretical learning)	Understanding of recorded video lectures shared by the facilitator	9
Step 3 (Co-creation goal setting and action planning)	Regarding the action plan developed with the facilitator as the team and leader, how much did you personally understand and agree with it?	9.57
	Did you grasp the importance of TFL and task-involving climate during the online video conference with the facilitator?	9.14
Step 4 (Action implementation)	Extent to which leadership behavior plans were implemented	7.86
Steps 5 and 6 (Daily monitoring, collective reflection, weekly monitoring, replanning goal and action)	Usefulness of the messaging app and the cloud-based survey system for leadership enhancement	8.43
	Usefulness of the leadership-focused online video conferences	9.29

Mean scores are based on a 10-point scale. Open-ended comments described experiences related to each step and the perceived benefits of periodic review processes. TFL, transformational leadership.

#### 3.2.1 Step 1: self-awareness

In this phase, participants completed a questionnaire aimed at assessing their perceptions of their current state in terms of commitment, motivation, and goal orientation. The level of understanding of the questionnaire process was rated highly (M = 9.0). Open-ended responses indicated that the activity provided an opportunity for personal reflection, as illustrated by comments such as, “*I was able to recognize my own issues and organize my thoughts*” *(Athlete Leader)* and “*Although I initially expected it to be tedious, the act of verbalizing my thoughts turned out to be valuable*” *(Athlete Leader).* These responses indicate that Step 1 effectively facilitated introspective engagement and supported the development of greater self-recognition among captains and athlete leaders. The clarity of the questionnaire items received moderately high ratings (M = 8.86), although some participants perceived certain terms as ambiguous, suggesting the need for further refinement to ensure consistent interpretation across respondents. Ease of response was rated particularly highly (M = 9.86), with several participants highlighting that the use of self-awareness questionnaire via the cloud-based survey system and smartphone accessibility reduced both cognitive and logistical burdens.

#### 3.2.2 Step 2: theoretical learning

In Step 2, participants engaged in asynchronous theoretical learning through recorded video lectures developed by the first author. These lectures introduced key concepts related to TFL and task-involving climate in a structured and sequential format. Participants evaluated their understanding of the lecture content highly (M = 9.0). Open-ended comments suggested that the clarity of explanation and the step-by-step presentation style contributed to comprehension. For example, one participant noted that the content was “*easy to understand thanks to the clear and structured explanations*” *(Captain)*. The asynchronous format also allowed participants to progress at their own pace, which may have supported deeper processing of the material. The combination of conceptual clarity, self-paced learning, and relevance to participants' existing leadership roles likely contributed to the positive evaluations observed in this step. Overall, Step 2 appeared to play an important role in establishing a shared theoretical grounding before participants moved on to co-creation goal setting and action planning in Step 3.

#### 3.2.3 Step 3: co-creation goal setting and action planning

In Step 3, each participant collaboratively developed leadership action plans with the facilitator and other leaders. This step focused on translating the theoretical understanding from Step 2 into concrete and context-specific leadership behaviors grounded in SDT. Understanding and agreement with the co-created action plans was rated highly (M = 9.57). Participants emphasized that being able to set their own goals within the collaborative process enhanced their sense of ownership. For example, one participant stated, “*By incorporating my own ideas alongside the facilitator's, we established an appropriate goal for team building*” *(Athlete Leader).* Another commented that because the goals were created through collective discussion, “*they were easy to understand*” *(Athlete Leader)*. These responses suggest that co-creation approaches supported both cognitive clarity and psychological investment. Participants also reported gaining a deeper understanding of TFL and task-involving climate during discussions (M = 9.14). For instance, one participant indicated that they were able to “*break leadership down into its constituent parts*” *(Athlete Leader),* while another highlighted a growing awareness that “*each member must fulfill a role aligned with their strengths and characteristics*” *(Athlete Leader).* These reflections suggest that Step 3 facilitated perspective-taking and encouraged participants to approach leadership as a distributed and context-adaptive practice within the team environment. Overall, Step 3 appears to have played a key transitional role by linking conceptual knowledge to practical leadership planning. The process fostered shared understanding of leadership direction and increased alignment between leaders' values and their action strategies.

#### 3.2.4 Step 4: action implementation

Step 4 centered on enacting the leadership behaviors defined in Step 3. Participants evaluated the extent to which they implemented their planned behaviors at a moderate level (M = 7.86), indicating partial but not full translation of intentions into consistent action. One participant described the challenge of sustaining conscious engagement, noting that leadership behaviors required ongoing awareness: “*I had to stay highly conscious, or I would forget*” *(Athlete Leader)*. This suggests that behavioral change required continued attentional effort rather than occurring automatically. At the same time, participants recognized that having clearly defined goals supported behavioral enactment. For example, “*Clear goals helped in translating plans to action*” *(Athlete Leader)*, suggesting that goal clarity functioned as a scaffold for initiating new leadership behaviors. The variation in responses indicates that while participants generally understood the expected behaviors, situational demands (e.g., training intensity, interpersonal dynamics, emotional regulation) influenced implementation consistency. Taken together, Step 4 highlights both the potential and the challenge of shifting leadership behavior in real-time team contexts.

#### 3.2.5 Step 5: daily monitoring and collective reflection

Step 5 involved daily monitoring and collective reflection through the messaging app. Use of the messaging app (and, in conjunction with Step 6, the cloud-based survey system) for ongoing reflection and communication received moderately high evaluations (M = 8.43). Participants indicated that articulating thoughts in writing promoted deeper reflection. Others commented that the messaging app for Step 5 “*enabled continued discussion beyond meetings*” *(Athlete Leader),* highlighting the value of multi-modal communication in sustaining engagement, though some noted that “*text alone made it difficult to convey nuances*” *(Athlete Leader)*. Overall, the messaging app contributed to information sharing and collective reflection; however, it was recognized that the messaging app alone could not shoulder the entire burden of reflection and monitoring, serving instead as a complementary and supplementary tool within the broader program structure.

#### 3.2.6 Step 6: weekly monitoring, collective reflection, and replanning goal and action

Step 6 involved periodic online collective reflection video conferences with weekly monitoring via the cloud-based survey system and the iterative adjustment of leadership action plans. The usefulness of these leadership-focused video conferences was rated highly (M = 9.29). Many participants emphasized that structured reflection allowed them to examine leadership from perspectives not typically discussed in daily training. One participant described gaining “*a new perspective through these discussions.*” *(Athlete Leader).* Another noted that the facilitator's style of facilitation “*praising our opinions and asking what we should do next*” created “*a positive and constructive atmosphere*” *(Athlete Leader),* supporting psychological safety and open dialogue. Collective introspection led to the acquisition of new perspectives and spurred subsequent action, and by respecting participants' actions and opinions, the meetings were conducted in an overall positive atmosphere.

### 3.3 Thematic analysis of post-program interviews

Inductive analysis of 29 transcripts (captains = 8, athlete leaders = 14, and coaches = 7) produced seven inter-related themes. Across themes, richer examples and more abundant case illustrations were obtained from the intervention group than from the brief intervention group.

#### 3.3.1 Theme 1: challenges before the program

Before the program began, leaders experienced challenge in coordinating perspectives and maintaining a shared understanding of how the team should function. Athlete leaders described uncertainty regarding their leadership identity, noting that although they carried responsibility, they lacked a clear framework for guiding their behavior. They explained that training sessions often proceeded without a clearly defined intention or focus, which caused emotional fluctuations in response to external conditions such as weather or field quality. “*I had lost sight of what kind of leader I should be and how I should act.*” *(Athlete Leader),* “*The atmosphere would deteriorate on rainy days.*” *(Athlete Leader)*

Captains reported that they tend to internalize responsibility and problem-solving, often acting alone even in areas where they felt less confident. This individual burden sometimes resulted in reactive communication when mistakes occurred during practice. “*I was always doing everything on my own.*” *(Captain)*, “*We would reprimand others harshly out of urgency.*” *(Captain)*

Coaches noted that leadership tended to be concentrated almost exclusively in the captain, creating a structure where responsibility and influence were not shared among leadership members or teammates. “*The team tended to be led solely by the captain.*” *(Coach)*

Taken together, leadership prior to the program was characterized by individualized responsibility, limited collaboration, and low structural support.

#### 3.3.2 Theme 2: collective reflection

Following the messaging-app exchanges and online video conferences with the facilitator and the athlete leadership group, most leaders reaffirmed goal alignment and perceived competence. Connecting TFL and task-involving climate factors to team conditions clarified individual roles, and sharing this process enhanced mutual support. Participants described discussions with fellow leaders as instrumental in achieving alignment of intentions. Even in the brief intervention group, the program allowed leaders to clarify team direction. “*Talking with fellow leaders helped us align our intentions.*” *(Athlete Leader)*

In the intervention group, the weekly use of the cloud-based survey system for self-evaluation and goal-settings provided a concrete mechanism to track progress over time. Collective reflection in the intervention group linked more directly to new actions. “*By recording goals and reflecting, we realized how to proceed next time.*” *(Athlete Leader)*

Captains reported a shift in their conceptual understanding of leadership from doing everything themselves to intentionally distributing responsibilities based on individual strengths. “*I realized it's better to leave what I’m not good at to others.*” *(Captain)*

#### 3.3.3 Theme 3: new behaviors

Through online video conferences conducted after undergoing self-awareness and theoretical learning, leaders were prompted to initiate new practices, such as individualized check-ins and expanding communication channels. Leadership shifted from directive and reactive to proactive and relational. In the brief intervention group, leaders themselves initiated new actions. “It w*asn't just about making a sound; I was able to create it while really considering how the listener would feel.*” *(Athlete Leader)*

Furthermore, in the intervention group, leaders created opportunities for all players to generate new actions. Participants also highlighted the importance of informal relationship-building, such as discussing rugby outside of training. “*We began to gather informally outside practice to discuss about rugby and share reflections.*” *(Athlete Leader)*

As captains, they facilitated information sharing and created opportunities for each player to demonstrate leadership. “*We created six messaging app groups and shared our analysis results within each group. We also informed the third-year students that the presentations would be led primarily by first and second-year students.*” *(Captain)*

Coaches observed that leaders increasingly functioned as intermediaries between players and coaching staff. Participants started to proactively voice their requests to the coach. “*They conveyed requests and coordinated timing between sessions.*” *(Coach)*

#### 3.3.4 Theme 4: outcomes and impact

In the brief intervention group, participants described perceived improvements in team cohesion and resilience during competitive matches. “*Even when conceding points, we encouraged each other and enjoyed until the end.*” *(Athlete Leader)*

In the intervention group, some participants self-reported performance changes (e.g., self-reported increased lineout success from under 50% to over 80%) and described the effects of their own behavioral adjustments and their perceived impact on the team through sustained actions. Athlete leaders reported approaching competitions with greater confidence, while senior-junior relationships were described as becoming stronger through shared responsibility and communication. “*When the season begins, that vague sense of unease has disappeared, and personally, I've come to be able to approach matches with quite a positive feeling.*” *(Athlete Leader),* “*My mindset shifted towards valuing coaching juniors more highly. The vertical bonds grew stronger through action.*” *(Athlete Leader)*

Captains reported gaining mental space to observe the team more objectively. “*Leaving energizing to others gave me space to observe the team.*” *(Captain)*

Coaches described more autonomous communication and more balanced dialogue between players and staff.

“The communication between players themselves, and their ability to exchange opinions firmly with the coaches, can be seen as both the result of the coaching side's implementation and this program's outcomes. It was beneficial having both our interventions and this program in place.” (Coach)

Given the feasibility design and reliance on interview accounts, these outcomes are presented as participant- and coach-perceived changes rather than evidence of causal effects.

#### 3.3.5 Theme 5: lessons learned

Initially, one athlete leader felt stressed about behavioral change, but it also led to learning about its effects of it. Even in the brief intervention group, there were lessons to be learned. “*I found it quite tough at first because pushing myself harder than usual inevitably led to fatigue. But once I realized that this boosted the team's strength, I found I could keep going without it feeling like such a struggle.*” *(Athlete Leader)*

In the intervention group, planning, action implementation and collective reflection cycles became established, and leaders reported learning the importance of reflection cycles to pause, reconsider situations, and actively incorporate alternative viewpoints. “*Whether I'd managed to achieve them this week, and considering whether I might have regressed since then, this process itself contributed to my growth.*” *(Athlete Leader),* “*After the program, I've started to pause and reconsider things. I've come to realize that looking at things from a different angle and seeking others’ opinions is quite important.*” *(Athlete Leader)*

Captains emphasized that leadership did not require perfection, nor did it necessitate handling every situation alone; rather, it involved co-creation team direction with others. “*The process of objectively gathering diverse perspectives from others through meetings and similar forums, and analyzing the team from a bird's-eye view, is an essential learning experience.*” *(Captain)*

Coaches highlighted that the most meaningful outcome was not only behavioral change, but the development of a shared team identity rooted in the process itself. “*The process leading up to the match was fulfilling, regardless of the final result.*” *(Coach)*

#### 3.3.6 Theme 6: challenges and suggestions

Participants and coaches noted that the duration and timing of the program limited the depth of behavioral stabilization regardless of the intervention period. “*Mentally, it felt like the tournament had arrived almost immediately. So, I thought that by starting around the beginning of the summer holidays, we could build up our concentration and the morale of the whole team, and then peak for the final tournament.*” *(Athlete Leader),* “*It's more effective to start a little earlier than the final few months.*” *(Coach)*

One participant reported difficulties in adjusting schedules and securing sufficient time to engage in the program. In addition, enthusiasm for the program was also identified as an area for improvement. “*Finding free time (to have an online video conference) was surprisingly difficult.*” *(Captain),* “*At first, I wasn't very familiar with the program. I think I could have used it more actively and effectively.*” *(Athlete Leader)*

#### 3.3.7 Theme 7: future prospect

Players and coaches suggested including younger leaders earlier, regardless of group type. “*If we include one representative from each year group… the message would spread more quickly.*” *(Athlete Leader),* “*It might be beneficial for potential future leaders to already be participating in the program.*” *(Coach)*

In the intervention group, the necessity for further meetings between leaders was suggested, and most of them expressed interest in expanding the program to other schools (considering the timing of intervention). The program was also seen as beneficial for off-field personal development.

“*It would have been better to increase opportunities for reflection in daily life, such as regarding the team's situation or before entering meetings.*” *(Athlete Leader),* “*It would be interesting to have sessions where leaders from each school could join and discuss matters.*” *(Captain)*

Coaches also expressed a desire for more frequent updates to check the process of this intervention and support leaders. “*There were moments when I considered how best to foster better connections with the players among three (facilitator, leaders, me). However, they were being treated so well, and their desire to change was so evident, that I decided to leave things as they carry on as usual.*” *(Coach)*

## 4 Discussion

This feasibility and acceptability study developed and implemented a leadership development program using co-creation approaches for an athlete leadership group. Rooted in TFL, task-involving climate principles, SDT, and shared leadership, the program was designed to address documented gaps in adolescent leadership education, namely the lack of structured training, insufficient opportunities for role clarification, and limited mechanisms for self-awareness and collective reflection. Consistent with these aims, both captains and athlete leaders judged the program feasible and acceptable, indicating that this study design may deliver developmentally meaningful leadership learning within the constraints of high school sport environments.

Across both brief and intervention formats, leaders successfully co-created TFL and task-involving climate-based action plans informed by their self-awareness, prior theoretical inputs, and online video conferences with the facilitator and the athlete leadership group. Even among those in the brief intervention group, these processes which aligned with individual preferences and shared understandings, were perceived as effective in initiating attitudinal and behavioral adjustments. However, groups exposed to repeated iterative cycles of planning, enactment, and collective reflection demonstrated more multifaceted development. This differentiation mirrors prior work showing that adolescent leaders require structured training opportunities (16), that systematic reflection and debriefing are foundational to leadership growth (51), and that behavior-focused, theory-guided support can meaningfully shape motivational climate (62–64).

The fully online delivery, combining synchronous sessions via an online video conference system and asynchronous coordination through the messaging app and the cloud-based survey system, was perceived as practical and familiar to participants. This format reduced logistical constraints and enabled timely micro-adjustments between sessions. In line with previous research, text-based updates facilitated daily progress monitoring and issue resolution, while live online video conference allowed leaders to surface shared situational awareness, jointly co-create action plans, and cultivate reciprocal support. Together, these mechanisms were described by participants as scaffolding a shift from individual, reactive leadership toward shared, proactive, and relational practices, aligning with theoretical expectations of TFL and shared leadership models. Participants' recommendations to include younger teammates earlier in the process indicate perceived developmental value and the potential for sustainable integration into school sport calendars. Taken together, these findings suggest that the current program is a potentially replicable model for adolescent athlete leadership development in elite high school rugby teams.

### 4.1 Co-creation approaches

Before the program began, each leader reported feeling anxiety and uncertainty about their role. The importance of constructing one's own leadership style had been pointed out (51), and the program's early phases, including self-awareness and brief theoretical learning appeared to help athletes clarify their motivation and leadership identity. The clarification of previously ambiguous roles through the online video conferences with the facilitator and the athlete leadership group was reported to contribute to the delegation of captain's responsibilities and the redistribution of duties. This process was described as bringing about behavioral changes among the leaders themselves and as fostering mutual support. As for each leader's key action, determining them collaboratively with other leaders and the facilitator allowed for the incorporation of diverse perspectives compared to individual decisions. This was perceived to result in more engaging and practical plans, supporting leader motivation. Furthermore, since each leader participated in the decision-making moments for other leaders' new actions, their sense of ownership as stakeholders may have increased. Regarding behavioral change, because their own intentions underpinned the new actions, they reported being able to implement them swiftly. Sharing their actions within the athlete leadership group was described as reducing concerns about behavioral change. These choices and actions map onto SDT and resonate with Generation-*Z* values (66), as well as rugby's strengths-based ethos (26). In addition, this pattern of self-reflection and explicit role division appears to support self-awareness, empathy, and interpersonal learning in youth leaders (69).

### 4.2 Theory-Driven approaches

Although the curriculum addressed all six components of TFL, allowing each leader to select one focal TFL behavior proved both engaging and feasible. The participants most frequently chose idealized influence, inspirational motivation, fostering acceptance of group goals, and individual consideration, these patterns broadly consistent with distributions observed in prior cricket and rugby studies (48, 49). In contrast, intellectual stimulation and high performance expectations were selected less often. A plausible interpretation is that adolescent leaders in relatively homogeneous squads may initially prioritize self-improvement and relationship building over peer-challenging behaviors. This aligns with Duguay et al. (64) who similarly found limited change in intellectual stimulation due to its association with coaching or mentoring responsibilities rather than peer leadership. Importantly, action plans were framed not only at the level of individual TFL behaviors but also to explicitly promote task-involving climate (improvement, effort/skill development, relatedness support). Therefore, even brief intervention exchanges that included non-leader teammates (e.g., encouraging players until the end) operationalized this climate focus. In addition, because action plans were theory-based, participants could more easily consider subsequent steps after completing a task and transition smoothly to the next cycle. Future iterations of the program should test whether earlier-season initiation and a longer intervention dose increase uptake of the less-selected TFL facets (e.g., intellectual stimulation) and strengthen task-involving climate processes and be associated with measurable.

### 4.3 The cycle of action implementation, collective reflection, and replanning goal and action

Regarding the program duration, we observed two distinct exposure patterns (brief intervention group vs. intervention group), which allowed us to examine the extent to which daily collective reflection and replanning promoted behavioral change among leaders. Even in the brief intervention group, simply setting up a single online video conference between the athlete leadership group and the facilitator to objectively assess the current situation, establish a shared understanding, and clarify roles together with collective reflection via the messaging app, was associated with participant-reported effects, and participants described immediate behavioral changes were observed. Specifically, the quality of encouragement was described as refined, and teams persevered until the end, even in seemingly unwinnable matches. Opportunities for communication among leaders also were reported to increase, with perceived ripple effects on players and coaches. However, the limited number of opportunities for collective reflection and adjustment appeared to constrain the consolidation and diffusion of these changes throughout the entire team. In contrast, the intervention group, supported by the cycle of multiple touchpoints (online video conferences, the messaging app interactions), were described as showing more stable enactment of leadership behaviors and deeper peer involvement. A notable development was the shift from leader-centric action to broader team participation: leaders' initiatives encouraged teammates to identify and implement their own leadership behaviors, with perceived strengthening of relational ties. Informal interactions (e.g., pre-practice conversations, small-group video analysis) reinforced formal learning, consistent with research on the developmental role of incidental dialogue in athlete learning environments (26). Weekly reviews were reported to enable leaders to evaluate progress collectively, integrate diverse perspectives, and revise action plans, which participants linked to increased confidence and behavioral consistency. These patterns align with documented mechanisms of team resilience with shared leadership, team learning, social identity, and positive emotion, highlighting the importance of dialogue, feedback, and psychological safety in athlete-driven leadership development (27). In sum, the brief intervention group supported initial awareness and role clarification, whereas sustained, multi-touchpoint engagement in the intervention group was perceived to more robustly support behavioral development, expanded player-led action, and strengthened intra-team relationships.

### 4.4 Challenges in making leadership behaviors a habit

Similar to Voight (51), leadership behavior was reported to improve in this study, yet challenges remained in translating this into consistent behavior. Leaders often understood what they aimed to do, but struggled to enact their intentions automatically under training pressure or emotional load. The sustainability of these behavioral improvements depended on whether leaders could transition from conscious effort to habitual enactment. While role clarification and daily communication through the messaging app supported reflection and alignment, these strategies alone were insufficient to stabilize behaviors into habit. To further strengthen behavioral consolidation, structured reflection meetings may need to be paired with more deliberate in-practice action supports such as explicit prompts, behavioral reminders, and situational cues embedded in training routines. Establishing a support network that extends beyond coaches (43) and facilitators, and explicitly include student managers or non-leader teammates may increase accountability and provide reinforcement within the daily training environment. Finally, a longer program dose may be required for repetition, feedback, and adaptation sufficient to consolidate new leadership behaviors into everyday practice.

### 4.5 Limitations

This study has several limitations. First, as mentioned earlier, the intervention was brief which may be insufficient to detect sustained behavior change or durable shifts in team culture. Second, the sample was small and demographically homogeneous (33 male athletes from elite Japanese high school rugby), limiting generalizability to other levels, sports, age groups, and to female athletes. Third, outcomes relied primarily on self-report and process ratings (with complete stepwise data from a subset of seven leaders), constraining causal inference and inviting response bias; future trials should incorporate objective behavioral/team metrics (e.g., observation, performance markers). Fourth, coach involvement was not systematically quantified even though variability likely influenced fidelity and engagement; subsequent studies should measure coach behaviors and model them as moderators. Fifth, the single-arm, feasibility-focused design precludes causal attribution; cluster-randomized or stepped-wedge trials are warranted to estimate effects on team-level outcomes. Sixth, recruitment through professional networks may have preferentially included teams with higher readiness and interest, potentially limiting transferability to less-engaged settings. Finally, features of the Japanese high school sport context (tight team bonds, hierarchical traditions, intense seasonal schedules) may limit transferability; cross-cultural comparative studies are needed to distinguish context-specific from generalizable principles of youth leadership development.

### 4.6 Future directions

#### 4.6.1 Recommendations for practice

Although the pre-competition calendar may transiently enhance cohesion (56), it constrains structural and cultural change. Implementing the program earlier such as during the off-season or summer break, would create the temporal latitude required for broader behavioral shifts. Participants also perceived the overall dose as short relative to prior leadership programs [e.g., (46, 51)], reinforcing the need for longer, flexible formats that are integrated with school and competition schedules and co-designed with coaching staff to optimize continuity and depth of impact. Early phases warrant stronger, more structured onboarding. Some teams needed additional time to adapt, and early engagement with facilitators was uneven. In line with Voight (51), providing clear behavioral guidelines and expectations in advance can facilitate autonomous participation. Collaboration with coaches also requires calibration. The coach–captain relationship is highly influential (31), and coaches' feedback benefits several psychological factors in young players (70). Yet unrestricted coach access to all program content may inhibit open peer dialogue. A balanced approach that provides selective feedback and visibility for coaches while preserving athletes' psychological safety (19) may maximize both engagement and oversight. Strengthening coaches' own leadership [e.g., transformational behaviors, shared decision-making; (12, 71)] and providing a facilitation guide for interaction and feedback (72) could further amplify effects. The introduction of AI coaches is already under consideration (73), and collaboration with such systems could potentially enable further expansion of the program. To support leadership succession, earlier inclusion of younger athletes may establish a developmental pipeline for current and future leaders (31). Consistent with Gould and Voelker (43), interschool exchanges can broaden perspectives and stimulate innovation; neutral, structured spaces for peer-to-peer learning (e.g., leadership forums) are a pragmatic mechanism. Finally, to diversify stimuli and sustain long-term engagement, the program may also consider combining face-to-face interactions with online cycles (54) and extending leadership development to all team members (74), thereby fostering inclusive cultures and diffusing leadership responsibility across the team.

#### 4.6.2 Recommendations for future research

Future research should evaluate the program using designs that better support causal inference and clarify mechanisms, including longer follow-up, comparison conditions where feasible, and multi-informant or behavioral indicators alongside self-report and interview data. It will also be important to examine dose–response patterns (e.g., single vs. repeated cycles of Steps 4–6) and to test contextual moderators such as season timing, coaching involvement, and team culture. Replication across sports, competitive levels, and mixed-gender settings would further strengthen generalizability and inform scalable implementation.

## 5 Conclusion

This study developed and implemented a leadership development program using co-creation approaches for an athlete leadership group with high school rugby team captains and athlete leaders. Through structured processes of self-awareness, theoretical learning, co-creation goal and action planning, action implementation, iterative collective reflection, and replanning goal and action, the program was judged to have strong feasibility and acceptability. Participants reported clearer role understanding, improved collaboration among leaders, and perceived improvements in their leadership behaviors, team functioning, and psychological growth.

The findings highlight the value of intentionally fostering leadership within adolescent teams, particularly by clarifying role expectations, co-creating actionable leadership behaviors, and embedding regular collective reflection into team routines. These mechanisms appear especially relevant in cultural environments where leadership responsibilities often concentrate on a single captain, and they offer a practical pathway for promoting more distributed and relational forms of youth leadership.

The intervention provides a replicable and culturally responsive model for athlete leadership development in Japanese elite youth rugby teams. Future research should examine extended and earlier-season implementation, incorporate objective and team-level performance indicators, and evaluate the model across diverse sports and cultural contexts to strengthen evidence for its developmental impact.

## Data Availability

The original contributions presented in the study are included in the article/Supplementary Material, further inquiries can be directed to the corresponding author.
